# Atelectasis Pulmonum1Read before the Buffalo Pathological Society, July, 1890.

**Published:** 1890-09

**Authors:** Irving M. Snow

**Affiliations:** Lecturer on Diseases of Children, Medical Department University of Buffalo, Physician to Children’s Department Fitch Dispensary, etc.; 371 Porter Avenue


### Buffalo Medical and Surgical Journal

Vol. XXX. SEPTEMBER, 1890. No. 2.

### Original Communications.

### ATELECTASIS PULMONUM.[1]

By IRVING M. SNOW, M. D.,

Lecturer on Diseases of Children, Medical Department University of Buffalo, Physician to Children's Department Fitch Dispensary, etc.

These two words signify the imperfect expansion of the lungs. Atelectasis Pulmonum is commonly known as collapse of the lungs. Now, collapse of the lungs is an exceedingly frequent condition in infancy or childhood, and may be observed in two entirely distinct phases, viz., congenital and acquired.

#### CONGENITAL COLLAPSE.

1. The lung tissues may never be fully expanded, as in children who are born asphyxiated. Here the pulmonary tissue remains in the fetal condition and the atelectasis is congenital.

2. Collapse of the lungs is observed in older children when the pulmonary tissues have for some time executed their normal function. In these cases areas of lung tissue become consolidated and impervious to air, and return to conditions found in fetal life.

Now, when I speak of pulmonary collapse, I do not mean that a whole lung falls together or that an entire lobe becomes dense and airless. Pulmonary collapse makes its appearance in scattered foci generally throughout both lungs, and at those regions of the lungs where the air enters last. Collapse of lung tissue takes place in children when air ceases to enter a lobule or group of lobules.

Thus there are two distinct causes:

1. An obstruction in the bronchial tubes which prevents the entrance of air into the alveoli. Hence, we see collapse of the lung in new-born children, whose bronchial tubes are full of blood and amniotic fluid, or again in older children with bronchitis. They

1. Read before the Buffalo Pathological Society, July, 1890.

cannot cough up the inflammatory secretions past the glottis, hence the finer tubes are blocked with bronchial secretion.

2. The second cause of pulmonary collapse is weakness of the respiratory muscles. We see collapse of the lung in premature children, and in children exhausted by diarrhea, or any acute or chronic disease.

Very commonly these two influences operate together. There is secretion in the bronchial tubes, which cuts off the air. The child cannot dislodge the obstruction, because it is too weak to cough it up. Air can no longer enter the pulmonary lobules. The residual air is quickly absorbed by the blood, the pulmonary tissue falls together, and the air spaces become obliterated.

Now, the subject of atelectasis or collapse of the lungs is a very important one in childhood:

1. Because very often your first efforts in pediatric practice is to resuscitate a newly born infant.

2. Because collapse of the lungs is exceedingly frequent in later childhood. It is an almost invariable complication of any serious pulmonary inflammation — bronchitis, catarrhal pneumonia, and whooping cough; and, aside from respiratory diseases, children debilitated from any exhausting malady — diarrhea, indigestion, or typhoid fever — may suddenly exhibit alarming symptoms of dyspnea and respiratory failure, which are apparently due to pulmonary inflammation, but which are actually due to collapse of the lungs.

I will first refresh your memories concerning the asphyxia of newly born children — atelectasis where the lungs have never been fully expanded.

These children enter the world under very disadvantageous auspices. They are either prematurely born or have suffered from a delayed and difficult labor.

Asphyxia is the direct result of the inability of the infant to fill its lungs, and is occasioned by a congenital weakness or by a mechanical obstacle. We observe asphyxia neonatorum in feeble, debilitated infants after breach presentation, prolapsus of the cord, placenta previa, or tedious labors; anything, in fact, which prevents the free circulation of blood through the placenta to the fetal heart, may cause asphyxia. Now, as long as the child's blood is fully oxygenated by osmosis from the maternal blood, there is no stimulus to the respiratory centers in the medulla oblongata, and the child makes no effort to breathe; but as soon as the oxygen is cut off by pressure upon the umbilical cord, the child must use its lungs. Now, if the placental circulation is cut off while the child's head is still in the parturient canal, it sucks into its air-passages blood, meconium, or amniotic fluid, though very little air.

When the baby is born with its lungs unexpanded or full of fluids, it exhibits very few signs of life.

A baby which is normally developed will commence to cry and twist about as soon as it reaches the external air. Its lungs are thus fully inflated, and its skin, being filled with oxygenated blood, is of a brick-red hue. An asphyxiated child lies very quietly, gasping feebly for breath; sometimes not breathing at all. The skin is livid and purple, the pulse slow and weak, and it makes only languid movements of its limbs. This is the so-called asphyxia livida.

If your efforts at resuscitation are not successful, the pulse grows quick and almost imperceptible, the child becomes perfectly limp, all the muscles are relaxed. The complexion instead of being purple is pallid. This constitutes the second stage of asphyxia — asphyxia pallida. Unless you can get the child to fill its lungs, it will show convulsive movements and probably die.

These cases either improve or perish in a few hours. But by well-directed treatment you may often establish a kind of imperfect respiration in which the lungs are only partially expanded. Your little patient will lie very quietly in its mother's arms or in the cradle, breathing rapidly. Respiration is difficult and ineffectual. If you watch the movements of the chest you will observe that the anterior portion of the chest recedes in inspiration; that is to say, that where the ribs join the sternum there will be a deep groove when the baby inhales. The abdomen and sternum instead of being protruded sink inward during inspiration. Recession of the cartilaginous portion of the chest and epigastrium simply indicates that air is mechanically hindered from entering the air cells, and is as common in croup as in collapse of the lungs.

This symptom is explained as follows: The diaphragm contracts and enlarges the cavity of the thorax; the air passages are obstructed and cannot admit air to fill the vacuum. Thus, the upper part of the abdomen and the cartilaginous part of the thorax are sucked in by the pressure of the external air.

In addition to the rapid superficial breathing and recession of the chest, the baby remains cyanosed and very weak. It may be somnolent, sleep all the time, will not nurse, and have slight convulsive seizures. When you examine the lungs you will find dulness in the lower lobes, and will find certain areas in the lung where you can hear feeble respirations, or no breath sounds at all.

Now, these symptoms of dyspnea and cyanosis may continue in a child born half asphyxiated, or they may appear quite suddenly in a child two to three weeks old who seemed weak at birth, but otherwise quite normal. They are perhaps induced by slight bronchitis, by choking, or by exposure to cold. In both cases you have the same symptoms; the baby is short of breath, livid, and breathing quickly and imperfectly; the hands and feet are cold, the epigastrium is sucked in during inspiration. You find a pulsation below the sternum, due to distention and overwork of the right ventricle.

If you are called in for the first time, you are expected to explain the reason of the child's puny growth or sudden shortness of breath. It is not due to any considerable amount of bronchitis, for the temperature is normal or sub-normal, which would not be the case if there were acute pulmonary inflammation. At a first glance the livid and purple color of the complexion might cause you to suspect a malformation of the heart, but a careful examination of the lung, the discovery of dulness in the lower lobes, and feeble or absent breath sounds, will commonly prove that the cyanosis is due to imperfect lung functions or non-expanded lung tissue, and not to congenital cardiac disease.

Yet I am bound to state to you, that to decide whether cyanosis in the first few weeks of life is due to a malformation of the heart or to unexpanded lungs is often a difficult matter. In both cases the foramen ovale and ductus arteriosus are probably open; for, when the blood is hindered in its exit from the right ventricle through the lungs, as is the case in pulmonary collapse, it flows directly from right to left auricle through the open foramen ovale in very young infants, and indeed may force open that aperture if it be already closed. If extensive tracts of lung tissue are consolidated by collapse, the foramen and ductus may remain permanently open.

I will relate to you a case which illustrates the condition of a young infant suffering from atelectasis of the lungs:

The child was prematurely born and very feeble at the time of birth. During the first week the skin became very blue and it had several attacks of intense dyspnea.

At the age of three weeks its symptoms were as follows:

It was very much cyanosed about the face and extremities and very short of breath. An examination of the lungs showed extensive dulness in the posterior part of the right lung, between the scapula and spinal column. Over this area no breath sounds could be heard, only a few moist rales. The left lung was resonant and well filled with air. There was never any fever, only cyanosis and dyspnea. The child's ill-health was evidently caused by collapse of a portion of the right lung.

A healthy wet-nurse was procured, the bodily nutrition increased by breast milk and wine, and the child made a complete recovery.

[From Meigs and Pepper — Diseases of Children.]

### PROGNOSIS.

The prognosis of asphyxia in newly born children is fairly good. A vast number of these children, after good vigorous work, will begin to breathe deeply, and the danger is over.

When the symptoms of dyspnea and blueness continue, and the atelectasis seems to spread, the child is in great danger and may succumb to convulsions or intercurrent bronchitis.

### PATHOLOGICAL ANATOMY.

You will find abundant explanation of the diminished or deficient lung function in infants dying of atelectasis pulmonum, if you examine the lungs. The portions of lung which are unexpanded are commonly the lower lobes, the anterior edge of the lungs about the sternum, and the right middle lobe — in short, those regions where the air enters last. They are of a dark red or purple hue, and are depressed below the surface, for the consolidation of collapse involves a shrinkage in bulk. If you cut out a portion of collapsed lung and put it in water, it will sink. To the feel, it is firm, does not crepitate, as lung tissue filled with air does. Collapsed lung is not easily torn, nor can you thrust your finger into it, as you can in tissue softened by inflammation. Bronchioles clogged with mucus and amniotic fluid. The cut surface is smooth and exudes a bloody serum. Lastly, the whole of the collapsed portion of the lung may be reinflated, all of the air vesicles are filled, and the purple color changes into a bright red hue.

Generally the ductus arteriosus and foramem ovale are found open, as in fetal life.

### TREATMENT.

The treatment of asphyxia in newly born infants is undoubtedly familiar to you all.

The mouth is to be cleared of mucus, and you should at once make artificial respiration, either by the method of Sylvester by raising the arms to the head and back again to the sides, or by the swinging method of Schultze, grasping the child by the sides of the chest and the arms and swinging it up and down.

In the failure of these means external stimulation is of great value — hot and cold water or electricity.

When respiration is established, the baby should be wrapped in cotton wool or warm flannel garments and kept very quiet.

If the baby is very weak it should by all means be fed upon breast milk, and in addition given a little wine or brandy.

Cyanosis is best relieved by laying the child upon its right side; this position merely favoring a freer action of the heart.

When the lungs remain only partially expanded, in addition to warmth, breast milk and stimulants, you may administer tonics, as a few drops of Huxham's tincture or a solution of the citrate of iron and quinine. Warm baths, with mustard, should be regularly employed. They diminish the congestion of the lungs and cause the child to take long breaths.

Dyspnea and lividity from a sudden collapse of the lungs is treated with stimulation, spirits of ammonia, digitalis and heat. You must always remember that you have no inflammation to combat, only a feeble vitality.

### ACQUIRED COLLAPSE.

Collapse of the lungs in later years is called, in distinction to the congenital form, acquired collapse.

This condition is most interesting, because it was formerly confused with catarrhal pneumonia and capillary bronchitis, and was treated as an inflammation of the lungs. It is caused by the same factors as atelectasis of the lungs in early infancy, by obstruction in the smaller bronchial tubes preventing the entrance of air into the alveoli, and by weakness of the respiratory muscles.

Pulmonary collapse seldom appears, except in children that are congenitally weak, or in those who are exhausted by diarrhea or various acute diseases. It is very common in rickety babies. Here the tendency to pulmonary collapse is greatly favored by the weakness of the respiratory muscles, the softness of the ribs, and the great liability of rickety children to pulmonary inflammation.

You will, however, most frequently observe collapse of the lung in children who are suffering from bronchitis, catarrhal pneumonia, and whooping cough. The cause is purely mechanical. All pulmonary inflammations are accompanied by more or less secretion. The bronchial tubes are full of mucous, which often a feeble child cannot cough up and expectorate. In this case the smaller bronchial tubes become plugged with mucous, which the child cannot dislodge by coughing. Air ceases to enter the air-lobules, the residual air is absorbed by the blood, and the whole tract of lung supplied by the obstructed bronchioles becomes airless and consolidated. When the bronchitis is intense and the child very weak, extensive areas of lung may quickly become collapsed. Although collapse will spread rapidly through the lung, it may also, under favorable circumstances, very quickly disappear. By vigorous stimulation, or by an emetic, you may dislodge the mucous plug, the lung will again become permeable to air, and all of the physical signs of consolidation will pass away in a few hours.

Collapse of the lung makes its appearance in the course of bronchitis in about this fashion:

A child of two years has a temperature of 100°; respiration, 35; pulse, 120; and a frequent harrassing cough. On your next visit you may find the child much worse. The mother states that it has coughed badly in the night and seemed very short of breath. You observe that the child is bluish about the mouth, is breathing at the rate of 100 to the minute, has a quick pulse, and is restless and panting.

When you examine the lungs you find recession of the anterior portion of chest during inspiration, slight dulness along the back of the thorax, auscultation over the areas of dulness, you hear faint breathing or no breath sounds at all. Sometimes there is distinct bronchial breathing, caused by a mass of collapsed lung lying around a bronchial tube.

This sudden change for the worse is often quite perplexing.

The degree of respiratory distress is greater than would be accounted for by the amount of bronchitis present. You might suspect that a pneumonia had complicated the bronchitis, were it not for the fact that there is no rise in temperature. Instead of the child being flushed and feverish, it is cold and livid.

Here the acute dyspnea and cyanosis is caused by collapse of lung tissue, from accumulation of mucous in the lungs and violent, ineffectual coughing. The dulness and faint breath sounds are caused by the consolidation and obliteration of a number of air lobules.

Now, this clinical picture is by no means infrequent. You will often see it in children under eighteen months suffering from bronchitis, and I sincerely hope you will carefully examine your cases and not diagnosticate collapse of the lung as pneumonia.

Nevertheless, some degree of pulmonary collapse is the rule in catarrhal pneumonia. With almost every death from catarrhal pneumonia, the fatal issue is caused as much by collapse of lung tissue as by inflammatory consolidation. We may judge of the appearance of collapse in pneumonia when there is a great increase of dyspnea and evidence of spreading consolidation, without a rise in temperature. In catarrhal pneumonia, as new lobules are attacked the fever should increase, but here we have diminished lung function without a higher temperature.

The physical signs in catarrhal pneumonia with collapse are obscure and puzzling. Now, ordinarily in catarrhal pneumonia you hear a variety of moist rales, with roughened or slightly bronchial breathing. When pneumonia is associated with collapse the signs in auscultation are exceedingly variable. At one time you will hear moist rales, with bronchial breathing. Next day you will hear scarcely any breath sounds at all, and at the end of twenty-four hours you will perhaps hear roughened breathing again. It is the shifting of the signs of consolidation that marks the course of collapse in catarrhal pneumonia; lung tissue is quickly collapsed and quickly reinflated.

Whooping cough is exceedingly apt to be associated with collapse of the lung. At the height of the disease there is commonly some bronchitis and violent paroxysms of coughing.

If the cough is very hard and there be much inflammatory secretion in the lungs, a child is fortunate that has not some pulmonary atelectasis.

Sometimes the symptoms are quite trivial. A child coughs very hard and lies panting and cyanosed for a few hours.

More often collapse of the lung leads to a serious condition, as in the case which I will relate:

The child was a well-nourished, breast-fed baby of twelve months.

It had suffered for seven weeks with whooping cough, complicated by diarrhea. For three or four days the coughing spells had been very severe and left the child very much exhausted. One day at noon the child coughed very hard and soon after became unconscious. I saw the child at two P. M. It was lying limp upon its mother's lap. Respiration, 80 to the minute; pulse, 140; temperature, 99°.

I examined the chest and found slight dulness in the right axillary region; over the rest of the chest the resonance was normal; ausculta-tion revealed faint breath sounds with distant bronchial breathing in the right axilla. In the left lung respiration was rough and harsh, while the vocal fremitus was equal on both sides.

There was no cardiac murmur.

The child was pallid and cold, with slight blueness about the mouth. I diagnosticated collapse of the right lung.

Next day the temperature, 99; pulse, 140; the breathing was rapid and very irregular, approaching the Cheyne-Stokes rhythm.

On the fifth day temperature and respiration were about the same. The pulse was weaker. There were now coarse creaking rales heard in both lungs. I discovered dulness in the right chest, both in front and behind. During inspiration there was retraction of the epigastrium; child coughed violently. I inferred that more of the lung tissue was collapsed and that the child had now a bronchitis. The child showed no improvement until the eighth day, when the dyspnea subsided some-what. The respiration sank to 60 to the minute; pulse, 130; temper-ature, normal.

Both lungs were full of creaking rales, but I could now hear the respiratory murmur in the right lung.

The baby appeared hungry and nursed very well. During the ill-ness two molar teeth made their appearance.

The baby now made a rapid and complete recovery.

This was a typical case of collapse of the lung during the course of whooping cough. Two very interesting features were the intense dyspnea and the total absence of a febrile temperature. All cases do not run so favorable a course. Nearly every fatal case of whooping cough is associated with collapse of the lungs. It must be remembered that more deaths are returned each year from pertusis than from typhoid fever.

The prognosis, with the acquired form of pulmonary collapse, is always serious:

1. Because it always occurs in weak and exhausted children.

2. Because it is commonly a complication of acute pulmonary disease where the breathing function is already badly impaired. When collapse occurs, large tracts of lung are rendered practically useless.

Hence, pulmonary collapse always increases the dyspnea and adds greatly to the dangers of heart failure, by causing distention and exhaustion of the right ventricle of the heart.

### PATHOLOGY.

The pathology of the acquired form of lung collapse is about the same as the congenital atelectasis. It is important to distinguish the condensed lung tissue of collapse from the inflammatory consolidations of catarrhal pneumonia. With collapse there is no pleurisy, a frequent complication of pneumonia. A collapsed lung can be reinflated with air, while lung tissues glued together before inflation resist all efforts at inflation. Wherever there is collapsed lung, the surface of the lobe is blue or violet, and the cut surface of a pneumonic lung is granular, and plugs of exudation can be squeezed from it. Collapsed lung when incised looks like muscle, and only bloody serum can be squeezed out of it.

Foci of collapse may be scattered all through the lungs, but its favorite seat is on the lower lobe, or on the anterior edge below the sternum. When much of the lung consolidates, there is commonly some emphysema.

### TREATMENT.

The treatment of the acquired form of collapse must vary as the condition is due to mere malnutrition, or exhaustion, or is caused by mechanical obstruction of the air tubes.

A child with collapsed lungs should be placed between flannel sheets, clothed in flannel garments, and kept in a warm, even temperature of 70 to 75°.

It must be nourished frequently and abundantly with breast milk, if an infant; older children, with milk and easily-digested broths. Reed & Carnrick's liquid peptonoid is an excellent thing to give. It contains digested beef essence, alcohol, and a small amount of farinacious food. Stimulants should always be administered, either brandy, wine, Huxham's tincture, or elixir of calisaya.

When collapse of the lung is a complication of bronchitis or of pneumonia, you must stimulate and give remedies which liquify the inflammatory secretions and aid in expectoration. These remedies are Hoffman's anodyne, spirits of ammonia, carbonate of ammonia, spirits of Mindererus, and syrup of senega. Emetics may sometimes be given with great advantage. When a child with bronchitis vomits freely, it sweeps out all of the mucous from its lungs, and may free the collapsed portions from their obstruction. Yet they are rather hazardous remedies, for emesis may be followed by a depression so grave that a child may not rally from it.

I believe that counter-irritation, persistently employed, is very serviceable in pulmonary collapse. Poultices of flour and mustard, or flax-seed poultices sprinkled with turpentine, are what I commonly employ. They should be applied fresh every three hours, and supplemented with warm baths.

I have now rehearsed the character of collapse of the lung in its various phases. I strongly urge you to familiarize yourself with its symptoms. A knowledge of pulmonary atelectasis, as seen in young children, will clear up many a puzzling case for you, and will explain many obscure and confusing symptoms in the pulmonary diseases of childhood.

371 Porter Avenue.

